# Stress of COVID-19, Anxiety, Economic Insecurity, and Mental Health Literacy: A Structural Equation Modeling Approach

**DOI:** 10.3389/fpsyg.2021.707079

**Published:** 2021-11-11

**Authors:** Yangxiu Hu, Baojuan Ye, Jiawen Tan

**Affiliations:** ^1^School of Psychology, Center of Preschool Education, Jiangxi Normal University, Nanchang, China; ^2^Center of Mental Health Education, Anhui Health College, Chizhou, China; ^3^School of Arts and Education, Chizhou University, Chizhou, China

**Keywords:** stress of COVID-19, economic insecurity, mental health literacy, anxiety, health promotion

## Abstract

The COVID-19 pandemic is currently a global health threat attributed to negatively affecting the mental health and well-being of people globally. The purpose of the current study is to examine the mediating roles of economic insecurity and mental health literacy in the relationship between stress about COVID-19 and anxiety. Results from the current study using a large sample of Chinese college students (*N* = 1,334) showed that stress of COVID-19 was positively associated with economic insecurity and anxiety while negatively associated with mental health literacy, which in turn was negatively associated with anxiety. These results elucidate our understanding of the role of mediators in stress about COVID-19 and anxiety. The findings are useful in terms of providing evidence for tailoring interventions and implementing preventative approaches to mitigate anxiety due to stress of COVID-19. Based on the present findings and within the context of COVID-19, the potential utility of promoting MHL to reduce the psychopathological consequences of COVID-19 is discussed.

## Introduction

COVID-19 has, for more than a year, forced a large portion of the global population to quickly transition to a new way of life (Peltz et al., 2020), which is a rapidly evolving global challenge and poses great risks to the global health (Chattu et al., 2020). Facing health threats, people have shown predictable threat responses such as anxiety, etc. (Shultz et al., 2015). COVID-19 as an unexpected epidemic, in addition to the physical impacts, has incurred significant psychological stress among those affected (Song, 2020). Studies demonstrated that negative effects of psychological responses (e.g., stress, anxiety, etc) affect the health and well-being of people suffering during the health crisis (Wu et al., 2005; Pappas et al., 2009). Anxiety, as one of the responses of COVID-19, was defined as a feeling of apprehension or dread accompanied with varied autonomic symptoms (Zhou et al., 2016). Anxiety involves a prolonged feeling of stress and worry, which makes it hard to cope with daily life (Lo et al., 2017). In order to provide appropriate mental health services and develop effective prevention and intervention strategies for people in response to COVID-19, it is critical to understand the mitigating factors associated with stress of COVID-19 and psychological problems like anxiety, etc. Thus, the purpose of the present study is to explore how stress of COVID-19, as a critical risk factor, influences anxiety, and to examine the potential mediating mechanism of economic insecurity and mental health literacy on the relationship between them.

### Stress of COVID-19 and Anxiety

Stress and anxiety are some of the key challenges for psychologists, psychiatrists, and behavioral scientists globally (Salari et al., 2020). Stress refers to an adaptive process that tends to show a variety of reactions when the internal and external environment is unbalanced (Folkman, 2013). Stressful life events, such as those instigated by the COVID-19 pandemic, have a significant influence on individuals' psychological function and well-being, and can be a catalyst for psychological problems including anxiety, etc (Ingram and Luxton, 2005; Arslan et al., 2020). Specific to stress of COVID-19, it is significantly related to greater anxiety symptoms (Limcaoco et al., 2020). Salari et al. (2020) also mentioned that stress of COVID-19 can increase anxiety, impair individual relationships, and lead to other negative consequences. Thus, we posit the following hypothesis:

**Hypothesis 1:** Stress of COVID-19 is positively related to anxiety.

Although research on past health epidemics has cited both increases in stress and mental and physical health symptoms (Brooks et al., 2020; Rajkumar, 2020), which have achieved fruitful results on the relationship between stress and anxiety a very prominent practical significance, little work has explored the potential factors that may impact the association between stress due to COVID-19 and global anxiety symptom severity (Manning et al., 2021). Therefore, within the context of COVID-19, the internal mechanism of the relationship between stress about COVID-19 and anxiety, and how the former influences the latter (mediating effect) is worthy of further discussion. In addition, few previous studies examined the mediating effects of variables like mental health literacy as individual factors on stress about COVID-19 and anxiety, and there is a lack of studies focusing on the impact of other factors related to the basic needs of individuals (e.g., economic insecurity due to COVID-19, as one of the manifestations of the unmet need for security), which is also consistent with the risk accumulation model which posits that behavioral symptoms (e.g., anxiety) are unlikely to be caused by a single risk like stress of COVID-19 (Li et al., 2016). Therefore, this study comprehensively investigated the possible mediating role of economic insecurity and mental health literacy between stress about COVID-19 and anxiety.

### Economic Insecurity as a Mediator

The need for safety/security is broadly defined as the need to feel safe from environmental threats and to perceive oneself as having sufficient material resources to ensure basic survival (Maslow, 1943). This broad need involves different facets (Maslow, 1970), including the need to have sufficient material resources for basic survival (i.e., economic safety) etc (Mani et al., 2013). The current COVID-19 pandemic is a rapidly evolving global challenge and like any pandemic, it weakens health systems, costs lives, and also poses great risks to the global economy and security (Chattu et al., 2020). Socioeconomic disparities resulting from job losses and other systemic barriers can also exacerbate mental health issues (e.g., anxiety, depression, etc) among the general population amid COVID-19 (Haidar et al., 2020). As one of the most common insecurities, economic insecurity (EI) refers to the sense of uncertainty and unpredictability generated by individuals related to their economic status (Abeyta et al., 2016; Chou et al., 2016; Losee et al., 2020), including fear of unemployment, feeling that the economic situation may get worse, etc (Kopasker et al., 2018). A sense of insecurity surrounding one's work life often stems from socioeconomic and personal characteristics of the individual, but insecurity may also be due to the historical context of the cohort to which one belongs (Wickrama et al., 2018). Clearly, the current COVID-19 crisis as a product of the specific historical context poses a threat to economic safety. Moreover, EI (Frankham et al., 2013) also comes with a psychological cost such as symptoms of anxiety and depression etc. With that in mind, what about the relationship among stress about COVID-19, EI, and anxiety?

Stress appraisal theory (Lazarus, 1999) contends that when an individual is exposed to a stressor, they subjectively appraise the threat and stressfulness of the stressor and assess available resources to manage the stressor. Accordingly, individuals who perceive their economic status as being insecure and anticipate a disruption to, or termination of, their economic status appraise the stressfulness and potential threat of economic insecurity (Wickrama et al., 2003). One important omission from recent studies, however, is an understanding of stress of COVID-19, or related stressors, in explaining exposure to economic insecurity. Furthermore, in recent years, studies have shown that EI can have a considerable negative impact on mental health (Clark and Georgellis, 2013). Rohde et al. (2013) found that EI and income decline or fluctuations are significantly related to individuals' emotional function and anxiety or other depressive symptoms, which had a negative and serious impact on individuals' mental health (Rohde et al., 2013; Van et al., 2015). Rohde et al. (2016) found that the negative effects of economic insecurity on mental health are much larger than the impact on physical health using data from Australia (Rohde et al., 2016). A study from developing countries found that individuals with EI had significantly higher stress and anxiety during COVID-19 (Salameh et al., 2020). Thus, we posit the hypothesis 2.

**Hypothesis 2:** EI is positively related to stress of COVID-19 and anxiety.

Sense of security, known as one of the basic needs, is a necessary nutrient element for the healthy development of individuals (Maslow, 1954). However, COVID-19 can elicit a lot of worry and insecurity in individuals (Brodeur et al., 2021), including uncertainty regarding one's health (e.g., Mertens et al., 2020) and economic concerns (e.g., Fetzer et al., 2020; Kleinberg et al., 2020). From a Maslowian perspective, when strong concerns for safety/security become salient, such concerns would play a preeminent role in individuals' functioning, leaving less room for other needs in the need-hierarchy, such as those studied in Self-Determination Theory (SDT), to play a supplementary role (Vermote et al., 2021). However, if the living environment cannot satisfy the basic needs, individuals may adapt poorly or switch to other resources to satisfy. From this point of view, the satisfaction of basic needs is not only the “outcome” affected by the environmental background, but also the internal “motivation” that drives the individual to make compensatory behaviors when the needs are not satisfied (Sheldon and Gunz, 2009; Sheldon et al., 2011). In other words, whether the basic needs are satisfied can be regarded as the key motivation mechanism of how environmental factors affect behavior. Consistent with this view, a large number of empirical studies showed that the satisfaction of basic needs not only played a mediating role between good environment (e.g., parental support, positive parenting, teacher support, etc.) and positive development outcomes (e.g., high well-being, high self-esteem, initiative and high academic achievement, etc.) (Taylor and Lonsdale, 2010; Vansteenkiste and Ryan, 2013), which also played a mediating role between adverse environment (e.g., adversity, high pressure, controlled parenting, etc.) and negative development outcomes (e.g., psychological distress, depression, anxiety and behavioral problems, etc.) (Vansteenkiste and Ryan, 2013; Corrales et al., 2016). Based on the above analysis, stress about COVID-19 may cause anxiety by preventing individuals from meeting their basic psychological security needs (i.e., avoiding the existence of EI) in real life. We expand on the extant literature by analyzing the effect of stress of COVID-19 on anxiety within a causal model that allows for variation of economic insecurity, which may be either objective or subjective in nature. However, few studies have directly examined the mediating role of EI on stress about COVID-19 and anxiety. In brief, by combining the above corollaries, in the context of the stress about COVID-19, we hypothesize the following association:

**Hypothesis 3:** EI mediates the effect of stress about COVID-19 on anxiety.

### Mental Health Literacy as a Mediator

Although the perspective of need satisfaction is helpful to understand the potential mechanism of stress of COVID-19 influencing anxiety, it is not comprehensive enough. Motivation is not only the “push” of internal needs, but also the “pull” of individuals' existing knowledge or cognitive concepts (e.g., mental health literacy etc.). Mental health literacy having received more attention in recent years has been found to be an important predictor of supportive attitudes toward mental health problems including anxiety, and toward help-seeking for the self and for others (Jung et al., 2017). Mental health literacy is also a central component to mental health support and development (Dang et al., 2017). This study will thus focus on the possible mediating role of mental health literacy on stress of COVID-19 and anxiety to a certain extent.

The term “mental health literacy” (MHL) was initially defined by Jorm as knowledge and beliefs about mental health disorders that aid in their recognition, management, and/or prevention (Jorm et al., 1997). Kutcher et al. later conceptualized MHL to include 4 domains: (1) understanding how to obtain and maintain good mental health; (2) understanding mental disorders and their treatments; (3) decreasing stigma against mental illness; and (4) enhancing help-seeking efficacy (Kutcher et al., 2016). Therefore, MHL addressed 3 inter-related concepts: knowledge (knowledge of mental illness and positive mental health), attitudes and help-seeking efficacy (Wei et al., 2015). Based on this, Jiang et al. defined MHI as “the knowledge, attitude and behavior habits that individuals develop in promoting their own and others' mental health and coping with their own and others' mental illness” (Jiang et al., 2021). Evidence shows MHL is a significant antecedents of mental health and has the potential ability to improve both individual and population health (Reavley and Jorm, 2012; Kutcher et al., 2015). High level of MHL (e.g., improved knowledge about mental health or mental disorders along with better awareness of how to seek help and treatment) may promote early identification of mental disorder risk and improve mental health outcomes (Lo et al., 2018). The literature above indicated that MHL may be a possible factor that plays a positive role in the promotion of mental health, as well as alleviating negative emotions such as anxiety and depression. Based on the analysis above, we posit the following:

**Hypothesis 4:** MHL is negatively related to stress of COVID-19, EI and anxiety.

According to the self-system process model (Connell and Wellborn, 1991), MHL can be used as a component of the individual self-system. Stress of COVID-19 as an external environment may influence development outcomes (anxiety, etc.) through individuals' self-system (e.g., cognitive concepts like MHL). A study of 800 college students by Han et al. (2012) found that mental health literacy was significantly negatively correlated with anxiety and depression scores. Brijnath et al. (2016) conducted a meta-analysis of 14 experimental studies on mental health literacy interventions from 2000 to 2015 and found that improving mental health literacy will improve mental health. Thus, we posit the following:

**Hypothesis 5:** MHL mediates the effect of stress of COVID-19 on anxiety.

This hypothesis is consistent with the basic idea of “situation-process-outcome model” (Roeser et al., 1996). The model points out that situational factors (such as stress of COVID-19) my impact the development outcome (e.g., anxiety etc) by influencing the psychological process (e.g., the acquisition of mental health literacy). The essence of this model is to explain the mediating process of situational factors on the development results.

### Integration of the Two Intermediary Mechanisms

The current study will also examine the mediating role of EI and MHL between stress of COVID-19 and anxiety. From a statistical point of view, the multiple mediation model has more advantages than the simple mediation model, which can determine the size of the total mediating effect, control one mediating variable (e.g., EI) and explore whether another mediating variable (e.g., MHL) has a significant effect. Meanwhile, it can reduce the parameter estimation bias caused by the neglected variables and the influence of other mediating variables. The relative size of different mediating effects can be compared as well (Preacher and Hayes, 2008). From a substantive point of view, the multiple mediation model can integrate the existing research, show the complementary mediation path and better understand the complex process as well as the mechanism of independent variables affecting dependent variables.

In this study, although both basic EI and MHL may explain the relationship between stress and anxiety, it is not clear whether the two variables work independently at the same time (parallel mediating effect) or first-to-then (chain mediating effect). On one hand, EI and MHL may act independently on anxiety (parallel mediation). On the other hand, the higher the degree of EI during COVID-19 in correlation with the lower the individuals' MHL, then the more EI and MHL may influence anxiety in a “serial” way (chain mediation).

There are obviously different practical meanings behind different intermediary models. If the chain mediation model is supported, sufficient intervention on one mediation variable can block the entire path from the independent variable to the dependent variable, meaning that intervention on the near-end mediating variables can be more effective than intervention on the far-end mediating variables. In summary, in view of the uncertainty of the relative relationship between EI and MHL, only exploratory analysis on these contents without proposing specific hypotheses is conducted here.

### The Present Study

**Figure 1:**
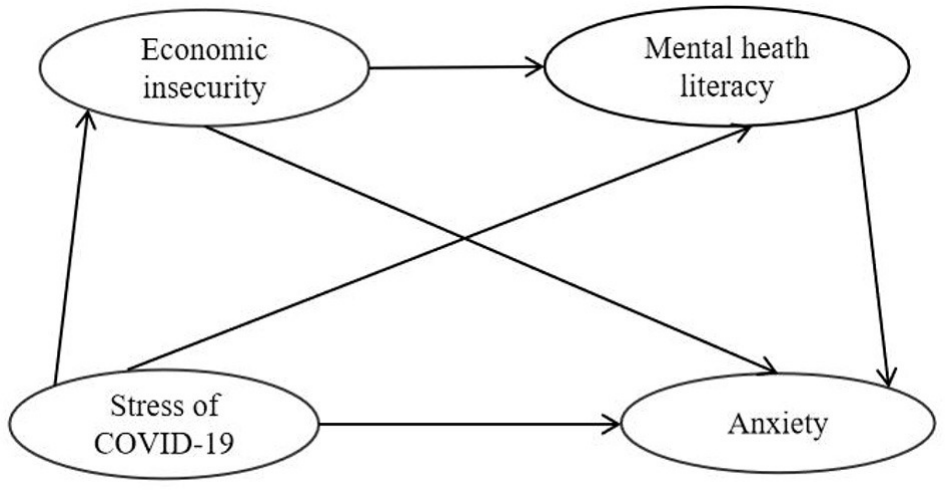
Model of the hypothesized mediating roles of EI and MHL in the relationship between stress of COVID-19 and anxiety.

Taken together, the current study first examined whether EI and MHL mediated the relation between stress of COVID-19 and anxiety (Figure 1). A structural equation modeling approach was conducted in this study by item parceling strategies (Wu and Wen, 2011). By parceling two or more items of the same scale into a new index, the composite score (total score or mean) was used as the score of the new index for analysis (Kishton and Widaman, 1994).

## Method

### Participants

The survey was approved by the ethics committee of the first author's university and all participants provided informed consent. A total of 1,368 Chinese college students participated from February 01–10, 2021 (e.g., the second little wave of COVID-19 in Hebei, China). After removing invalid observations (e.g., missing data or other errors), 1,334 participants (74.7% female) were included in the final analyses. The sample was composed of mostly students from rural areas (62.7%) while sub-urban (23.0%) and urban (14.3%) comprised the minority. The mean age was 20.02 (*SD* = 0.56).

### Research Instruments

#### Stress of COVID-19 Scale

The Coronavirus Stress Measure (CSM) was adapted from the 14-item perceived stress scale (PSS, Cohen et al., 1983) to assess COVID-19 related to stress (Arslan et al., 2020). The adapted CSM (Arslan et al., 2020) in the study included 5 items with scoring based on a 5-point Likert scale with a good reliability (α = 0.83), ranging between 0 = *never* and 4 = *very often* (e.g., “How often have you been upset because of the COVID-19 pandemic in the last month”) along with five items (standardized loadings ranging from 0.58 to 0.84) and a good validity (TLI = 0.960, CFI = 0.980, SRMR = 0.069). In the Cronbach reliability analysis, high reliability requires the value of alpha coefficient to be higher than 0.8, along with good reliability just requiring α being 0.7–0.8 (Eisinga et al., 2013). Responses to all items were averaged, and higher scores indicated higher levels of stress of COVID-19 with α = 0.701 in this study. All the data of exploratory factor analysis (EFA) and confirmatory factor analysis (CFA) on the four scales in this study were obtained through splitting the sample into random halves. By EFA here, KMO = 0.788, only one factor (5 items) with 2.506 > 1 of the eigenvalue was extracted and the variance interpretation rate after rotation of this factor was 50.124% > 50%. CFA also showed that all the factor loadings ranged from 0.436 to 0.708 and the one-factor model fit the data well: χ^2^*/df* = 1.553, TLI = 0.979, CFI = 0.990, RMSEA = 0.044, SRMR = 0.029, which substantiated that the adapted CSM could be used to measure stress of COVID-19 in this study.

#### Economic Insecurity Scale

The economic insecurity scale was used to measure economic insecurity during COVID-19 (Schwarz et al., 1997), which consisted of 8 items (e.g., “I often worry about whether I can afford my expenses”) across two dimensions: economic insecurity of parents and self economic insecurity, each with good reliability (α = 0.71 and α = 0.81) and the whole scale had a good content validity. Abeyta et al. (2016) used the scale with α = 0.88 and Li (2020) also used the scale with a good reliability (α = 0.76) among Chinese participants. All responses were measured on a 4-point Likert scale (1 = *Strongly disagree*, 4 = *Strongly agree*), with α = 0.788 in this study. Responses to all items were averaged, and higher scores indicated higher levels of EI. By EFA, KMO = 0.797, two factors with 3.457 (4 items) and 1.507 (4 items) of the eigenvalue were extracted and the variance interpretation rate after rotation of two factors was 62.303% > 50%. CFA also showed that all the factor loadings ranged from 0.541 to 0.801 and the two-factor model fit the data well: χ^2^*/df* = 2.400, TLI = 0.929, CFI = 0.952, RMSEA = 0.094, SRMR = 0.041.

#### Mental Health Literacy Scale (MHLS)

The “National Mental Health Literacy Questionnaire” (NMHLQ) compiled by Jiang was used to measure MHL (Jiang et al., 2021). NMHLQ consisted of 60 items (e.g., “When I feel bad and low energy, I know how to adjust myself.”) and included 6 dimensions pertaining to mental health literacy, mental illness literacy, self-help literacy, self-promotion literacy, help others literacy, and Promote others literacy. All items of the former two dimensions were measured on a 2-point Likert scale (0 = *False*, 1 = *True*), all items of the latter four dimensions were measured on a 5-point Likert scale (1 = *Strongly disagree*, 5 = *Strongly agree*). Refer to the original questionnaire (Jiang et al., 2021), the scoring method of the total is as follows. First, the 5-level score is converted into a binary score similar to *True* and *False* judgment, so as to make the scores of different types of questions equal. Second, 1 point for the “strongly agree” or “agree” option and 0 points for the other three options. The main measurement indicators of the original scale are as follows: the internal consistency reliability of the six sub-questionnaires is between 0.64 and 0.76; the test-retest reliability of the questionnaire at a 3-week interval is 0.72; all items are converted to 0, 1 scoring. The two-parameter Logistic model is used to analyze the degree of discrimination (80% item within a reasonable range of [0.5, 2]) and difficulty (88.3% item within a reasonable range of [−3, 3]), which indicates that the psychometric index and content validity of MHLS is pretty good (Jiang et al., 2021). After the unified dimension scoring in the current study, α = 0.891. Higher scores of the total indicated higher levels of MHL. By EFA on the six subscales of NMHLQ, KMO = 0.747, the only factor (6 parcels/subscales) with 3.094 > 1 of the eigenvalue was extracted and the variance interpretation rate after rotation of this factor was 51.566% > 50%. CFA on the six subscales also showed that all the factor loadings ranged from 0.458 to 0.783 and the model fit the data well: χ^2^/df = 1.517, TLI = 0.922, CFI = 0.953, RMSEA = 0.089, SRMR = 0.002.

### Anxiety Scale

Generalized Anxiety Disorder-7 (GAD-7) was widely used to measure anxiety, the internal consistency of which was excellent (α = 0.92) with all the factor loadings ranging from 0.69 to 0.81 (Spitzer et al., 2006). GAD-7 consisted of 7 items (e.g., “Feeling nervous, anxious or irritable”). Participants rated each item on a 4-point Likert scale (0 = *Never*, 3 = *Almost every day*), with α = 0.933 in this study. Responses to all items were averaged, and higher scores indicated higher levels of anxiety. By EFA, KMO = 0.928, the only factor (7 items) with 1.507 of the eigenvalue was extracted and the variance interpretation rate after rotation of the factor was 70.034% > 50%. CFA also showed that all the factor loadings ranged from 0.718 to 0.873 and the one-factor model fit the data well: χ^2^*/df* = 1.072, TLI = 0.995, CFI = 0.997, RMSEA = 0.033, SRMR = 0.009.

## Data Analysis

The first purpose of this study was to investigate the correlation between stress of COVID-19, EI, MHL, and anxiety. To this end, descriptive statistics and Pearson's correlational analyses were first conducted by SPSS 22.0. Tests of normality revealed that the study variables showed no significant deviation from normality (e.g., Skewness < |3.0| and Kurtosis < |10.0|; Kline et al., 2005; Drezner et al., 2010). The second purpose was to examine the mediation model on a structural equation model by AMOS 24.0. The model included four latent variables (stress of COVID-19, EI, MHL, and anxiety) that were made up of 20 parcels to reduce model complexity (Preacher and Hayes, 2008); the average scores for each parcel were used as indicators in the model. The model included a direct effect of stress of COVID-19 on anxiety and three indirect effects of mediation through EI and MHL: stress of COVID-19 → EI → anxiety; stress of COVID-19 → MHL → anxiety; and stress of COVID-19 → EI → MHL → anxiety. Missing data were estimated using full information maximum likelihood estimation, and robust maximum likelihood estimation was used to account for non-normality. Meanwhile, standardized regression coefficients were presented to quantify the strength of association between pairs of variables. The indirect effects of the mediation model were checked using procedures of bootstrapping confidence intervals (CIs) with 5000 random samples (Hayes, 2013). The model fit was evaluated using several common fit indices: χ^2^*/df*, TLI, CFI, NFI, RMSEA, SRMR, and RMR. The following were considered indices of good fit with severe standards: χ^2^*/df* <3 (not severe standard with 5), TLI > 0.90, CFI > 0.90, NFI > 0.90, RMSEA < 0.05 and RMR < 0.05 (Browne and Cudeck, 1992).

## Results

### Preliminary Analysis

The Means, *SD*s, and Pearson correlations are presented in Table 1. Stress of COVID-19 was positively correlated with EI and anxiety. MHL was negatively correlated with stress of COVID-19, EI, and anxiety. Gender was positively correlated with stress of COVID-19, EI, anxiety, and not with MHL, whether meaning that women perceived a higher stress and anxiety as well as having a higher level of EI or not. Except for the positive correlation of EI and stress of COVID-19, there was no correlation in MHL and anxiety on family locus, meaning that students from rural areas have a higher level of stress of COVID-19 and EI. It is necessary to further study whether this is related to the relatively poor economic conditions in rural areas. Here, all findings supported our given Hypotheses 1, 2, and 4.

**Table 1:** Descriptive statistics.

	***M***	***SD***	**1**	**2**	**3**	**4**	**5**	**6**
1. Gender	1.747	0.435	1					
2. Family locus	2.484	0.732	0.137^**^	1				
3. Stress of COVID-19	0.581	0.440	0.094^**^	0.055^*^	1			
4. Economic insecurity	0.511	0.324	0.056^*^	0.089^**^	0.215^***^	1		
5. Mental health literacy	0.521	0.064	−0.034	−0.013	−0.208^***^	−0.159^***^	1	
6. Anxiety	0.395	0.467	0.088^**^	0.020	0.576^***^	0.293^***^	−0.326^***^	1

*N = 1358.*

*** *p < 0.001;*

** *p < 0.01;*

* *p < 0.05.*

*Gender: 1 = male and 2 = female. Family locus: 1 = Urban, 2 = Sub-urban, and 3 = Rural*.

### Common Method Biases Test

Using the self-report method to collect data may lead to common method biases. Therefore, in the process of data collection, we carried out such corresponding controls as reverse questions for some items (Zhou and Long, 2004). By Harman single-factor testing, the results of EFA showed that all the eigenvalues of 5 factors were >1 (with the eigenvalues of 5.032, 2.212, 1.878, 1.452 and 1.206, respectively). The variation of the first factor was 25.161% (<40% of the critical standard) along with 58.944% of all the five factors. Thus, no significant common method biases existed in the measurement (Xiong et al., 2012).

### Measurement Model

A confirmatory factor analysis (CFA) was used to test the fit of the measurement model. Here, the above-mentioned four latent variables (stress of COVID-19, EI, MHL, and anxiety), with 20 parcels as indicators, comprised the measurement model. Results indicated that the data fit the model well: χ^2^*/df* = 3.362, TLI = 0.953, CFI = 0.964, NFI = 0.950, RMSEA = 0.042, RMR = 0.007. Further, all factor loadings on the latent variables were significant (*p* < 0.01), indicating that the latent factors were well represented by their respective indicators.

### Measurement and Structural Mediation Model

**Figure 2:**
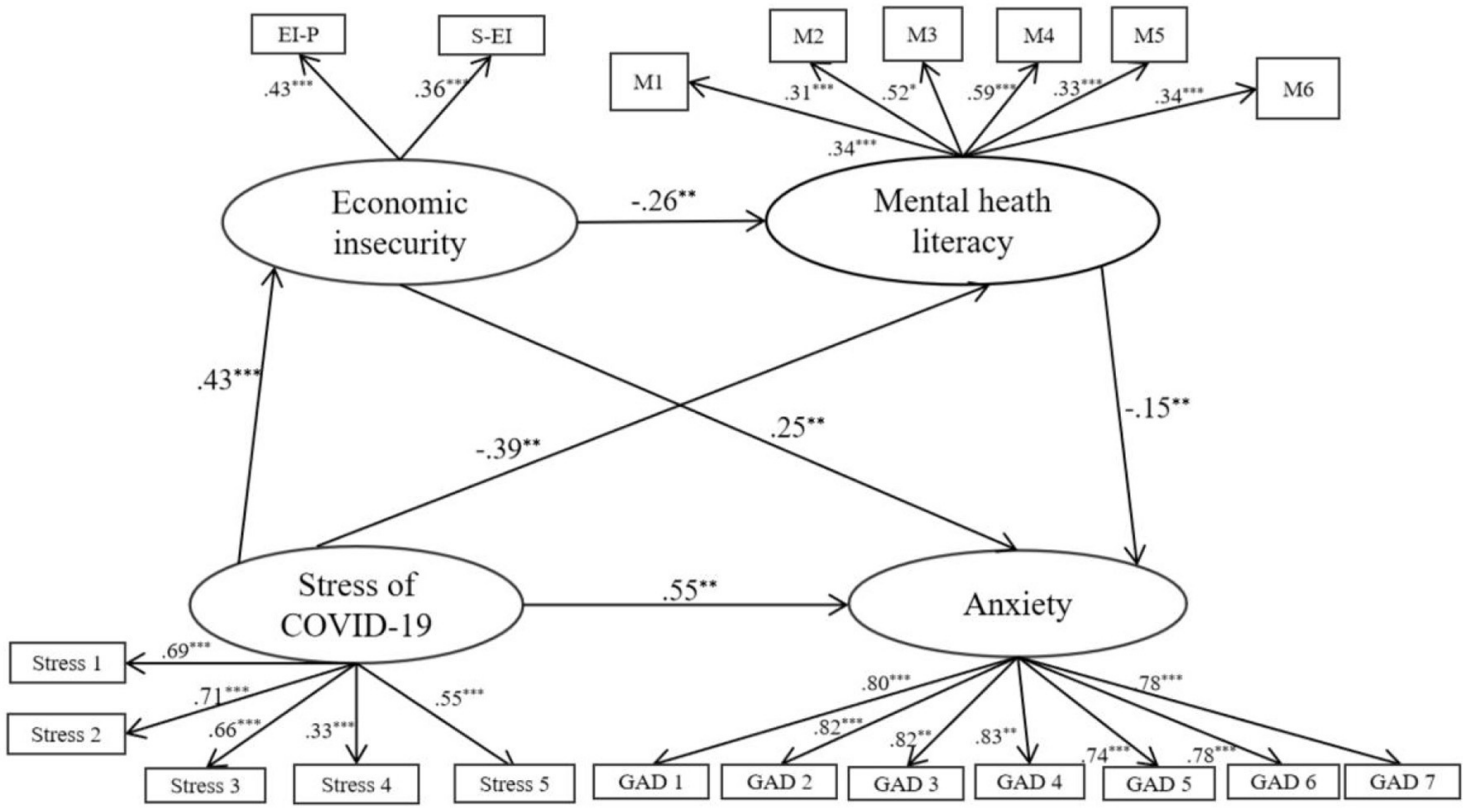
Structural equation model of the proposed relationships.

As shown in Figure 2 and Table 2, the structural equation model (modification based on MI > 20), which was used to examine the relationship among stress of COVID-19, EI, MHL, and anxiety fit the data well: χ^2^*/df* = 3.085, TLI = 0.959, CFI = 0.966, NFI = 0.951, RMSEA = 0.040, RMR = 0.007 (Fang et al., 2011). Analyses on the total indirect effects indicated that EI and MHL partially mediated the relationship between stress of COVID-19 and anxiety [γ = 0.183, SE = 0.078, *p* < 0.01, 95% CI (0.112, 0.337)]. Meanwhile, when examined separately, all the three indirect paths were significant: (a) stress of COVID-19 → EI → anxiety [γ = 0.106, SE = 0.099, *p* < 0.01, 95% CI (0.049,0.360)], (b) stress of COVID-19 → MHL → Anxiety [γ = 0.060, SE = 0.034, *p* < 0.05, 95% CI (0.001, 0.139)] and (c) stress of COVID-19 → EI → MHL → anxiety [γ = 0.017, SE = 0.034, *p* <0.05, 95% CI (0.004,0.055)]. The results showed that the two parallel mediating effects of (a) and (b) as well as the chain mediating effect of (c) were significant, meaning that both EI and MHL, respectively mediated the relationship between stress of COVID-19 and anxiety, along with the chain mediating effect of both EI and MHL. Consequently, the total effect of stress of COVID-19 through two mediating variables of EI and MHL on anxiety was up to 0.737. In addition, EI was found to mediate the relationship between stress of COVID-19 and MHL [γ = −0.111, SE = 0.084, *p* <0.01, 95% CI (−0.353, −0.026)]. MHL was found to mediate the relationship between EI and anxiety [γ = 0.040, SE = 0.050, *p* <0.05, 95% CI (0.008, 0.114)] as well. It is worth noting that when the bootstrap method is used to calculate the significance of the mediating effect, the 95% confidence interval should not include 0. Thus, all findings here supported our given Hypotheses 3 and 5.

**Table 2:** Standardized indirect effects of stress of COVID-19 on anxiety.

**Parameter**	**Indirect effect**	***SE***	***p***	**95% CI**
(a) *Stress → EI → Anxiety*	0.430 × 0.258 = 0.106	0.099	0.001	0.049, 0.360
(b) *Stress → MHL → Anxiety*	−0.390 × −0.154 = 0.060	0.034	0.046	0.001, 0.139
(c) *Stress → EI → MHL → Anxiety*	0.430 × −0.246 × −0.154 = 0.017	0.034	0.024	0.004, 0.055
(d) *Stress → Anxiety*	0.106 + 0.069 + 0.017 = 0.183	0.078	0.001	0.112, 0.337

*Stress = Stress of COVID-19*.

## Discussion

This study analyzed the effects of stress of COVID-19 on anxiety and extended the literature by investigating the potential mediating effects of EI and MHL in this relationship. The results supported our hypotheses about the significant effects as well as the direction of effects. As expected, stress of COVID-19, EI, MHL, and anxiety had significant relationships with each other. Stress of COVID-19 was significantly positively correlated with college students' EI and anxiety while negatively associated with MHL, which in turn was negatively associated with anxiety. Moreover, we found that EI and MHL mediated stress of COVID-19's effect on anxiety, and the indirect effect was significantly stronger via EI than via MHL. The study provided a unique opportunity to interpret the results within the context of the ongoing pandemic. Firstly, our results showed that females tended to report greater stress of COVID-19 and anxiety. As elevated levels of stress can increase anxiety, future research may be necessary to examine the extent to which females, compared to males, are at greater risk of their stressors from COVID-19 becoming debilitating than adaptive. Besides, by examining the relationship among variables, other meaningful findings were obtained.

### The Mediating Effect of EI

This study found that EI played a mediating role on the relationship between stress of COVID-19 and anxiety. In current study, EI as an important variable, was introduced to explain why a positive relationship between stress of COVID-19 and anxiety existed. Specifically, stress of COVID-19 may lead to the increase of EI, the result pattern of which conformed to the “gradient effect.” In other words, as the stress of COVID-19 increased, the level of EI increasing followed by. However, no critical value of stress of COVID-19 existed. After that, the obstacle of the increase of stress level to the sense of security deteriorated sharply (“positive acceleration mode”) or tended to be flat (“negative acceleration mode”). Although this “linear model” seemed to have little effect in explaining the “negative acceleration model” of stress of COVID-19 and anxiety, the high level of stress of COVID-19 did have a great impact on meeting the need of economic security.

Within the context of COVID-19, EI is a risk factor of anxiety. From the perspective of self-determination theory, stress of COVID-19 can make individuals' sense of security fail to be fully met, by which EI appeared (Li et al., 2016). Because one of the basic motivation of human beings is to seek the satisfaction of various psychological needs, sense of security, as one of the basic psychological needs, has its dynamic characteristics (Deci and Ryan, 2000). Under COVID-19, if individuals are unable to meet their basic psychological needs (such as the existence of EI) in the long run, they are likely to fail to make better cognitive adjustment and eventually lead to many negative emotions, such as anxiety etc. This is consistent with the diathesis-stress model which suggests that certain underlying vulnerabilities combined with stressful life events result in the development of mental disorders (Heim, 1999). That is, EI, as one of certain underlying vulnerabilities, interlocked with stress of COVID-19, increased anxiety. Briefly, EI played a mediating role on the relationship between stress of COVID-19 and anxiety.

Similar to prior research, stress of COVID-19 was positively associated with college students' EI (Vermote et al., 2021). Based on Maslow's prepotency principle, it can be expected that both in peaceful and stable conditions, such as during vacation periods, as well as in distressing and unstable conditions, such as during the COVID-19 crisis, these needs might play a predictive role (Maslow, 1970; Vansteenkiste et al., 2020). That is, during stressful times, the satisfaction of security needs would help to replenish one's resources, thereby fostering well-being, while simultaneously serving as a source of resilience and buffering against ill-being and maladjustment. In contrast, need frustration (e.g., EI) would create additional risk for mental health problems (i.e., more ill-being like anxiety) beyond the effect of felt uncertainty (Vermote et al., 2021).

Additionally, citizens' basic needs could potentially serve as a lever for mental health in times of threat (Laporte et al., 2021). Therefore, from a practical perspective, people receive ideally contextual support for their needs from others (e.g., family members and friends) to reduce EI (Clement et al., 2015). At a macro-level, eliminating individuals' EI also depends to some extent upon governmental policy and, in particular, the government's capacity to systematically use a motivating communication style such that citizens more willingly endorse the measures (Martela et al., 2021), while also taking sufficiently risk-reducing measures to keep citizens' feelings of EI and anxiety under control (Guido, 2015).

### The Mediating Effect of MHL

The current result demonstrated that the higher MHL predicts lower anxiety, which is consistent with the result of previous research conducted with Chinese participants (Ming and Chen, 2020), and it indicated that MHL is an important protective factor for anxiety. The diathesis-stress model, which also suggests that protective factors serve to mitigate the impact of stressful life events, can be used to explain this finding (Heim, 1999). In this regard, as a positive psychological quality, MHL can effectively help reduce psychological problems like anxiety due to stress of COVID-19. Thus, the current finding adds empirical support for the relationship model of MHL and mental health (Ming and Chen, 2020). The research results also support the “situation-process-results model” proposed by previous studies, that is, stress as a remote situational factor can promote the importance of MHL to recognize this relatively near-end psychological process, thereby promoting the reduction of anxiety. This finding can be explained from the perspective of social development models and social control theory. When MHL encourages individuals to develop the knowledge and ability to cope with stress effectively, individuals will strive to control emotions and behaviors, thereby avoiding the appearance of current psychological consequences such as anxiety (Hawkins et al., 2001). On the contrary, when individuals lack the self-cultivation to actively cope with stress of COVID-19, it may cause anxiety in many aspects, and even a series of problematic behaviors (Maddox and Prinz, 2003). All in all, MHL played a mediating role on the relationship between stress of COVID-19 and anxiety.

The most commonly used way to intervene or enhance MHL, from a practical perspective, is education and exposure (Arboleda-Flórez, 2008). Education aims to get rid of the myths related to mental diseases by providing information related to mental problems. It is generally carried out in the form of thematic lectures, courses, distribution of reading materials, group counseling, talks, etc (Liu et al., 2017). Exposure refers to exposing individuals to patients with mental illness, listening to patients with mental illness share their stories, and reducing individual stigma attitudes toward patients with mental illness by increasing empathy (Corrigan et al., 2015).

### The Chain Mediating Effect of EI and MHL

EI can damage mental health. Self-Determination theory (SDT) believes that EI may hinder individuals from meeting various psychological needs, thereby harming their mental health (Deci and Ryan, 2000). Some researchers used job stability as an indicator to investigate the impact of economic insecurity caused by job instability on mental health, and found that EI is negatively correlated with self-assessed health. In view of this, stress of COVID-19 can lead to possible EI firstly, then to hindering the use of self-help mental health literacy secondly; and last to influencing individuals' mental health as anxiety. In brief, the path of Stress → EI → MHL → Anxiety is effective. Therefore, in actual work, in order to alleviate the impact of stress of COVID-19 on anxiety, beside the overall social and economic recovery and development to reduce EI, a more operable and effective way is to promote the individuals' MHL. MHL has achieved a certain impact on mental health policies internationally. Many countries have launched improvement programs of MHL (Jorm, 2015). Studies in Australia, Canada, the United States, and Europe have shown that some specific interventions measures can effectively improve people's MHL (Jorm, 2015; Kohls et al., 2017; Sampogna et al., 2017). Specifically embodied in the following aspects: (a) Social intervention campaigns (by various paths of publicity and education to improve the public's ability to recognize mental illness), (b) School education intervention, (c) Self-service applications and (d) Mental health first aid training (Ming and Chen, 2020).

The COVID-19 pandemic introduced a complex worldwide stressor (Gruber and Rottenberg, 2020; Gruber et al., 2021). However, it is well known that not all individuals who perceive stress of COVID-19 develop affective disorders, with cognitive and emotional responses (like anxiety etc.) playing a critical role in determining whether anxiety follows such stress (Aldao and Nolen-Hoeksema, 2010). It will be important to pay attention to exaggerated perceptions of threat (which have been linked to anxiety). Especially for college students, the combination of mental health, financial, and social changes during COVID-19 also poses unique challenges (e.g., Arnett, 2004). Therefore, during the COVID-19 epidemic, the chain mediators found here may help to reduce anxiety from the perspective of EI and MHL.

## Limitations and Future Directions

Several constraints exist for the current study. The cross-sectional nature of the study limits causal inference and future research may utilize experimental and/or longitudinal designs to further test the given model. Secondly, due to the large sample size of this study, the variable measurement adopts the self-report method, which may cause method effect. In the future, more objective methods such as teachers' evaluation can be used to collect data. In addition, future research can also explore the mediating role and mechanism of other important variables (such as cognitive variables) between stress and anxiety. Thirdly, in view of the problem that the subjects are all Chinese college students, whether the research results can be extended to other groups remains to be tested. Fourthly, except for some of the participants who came from Hebei Province, where the second little wave of COVID-19 occurred in China at the beginning of 2021, the mean level of stress of COVID-19 and anxiety for the whole was not as high as expected, which may be related to their low risk of epidemic exposure. However, it is not contradictory to the existence of a positive correlation between stress of COVID-19 and anxiety. Fifthly, as the current study was conducted with a large sample of college students in China and more female subjects than men, whether the findings discussed above could be generalized to other groups remains to be determined, as well as a better future research design to come. At last, given the dynamic development of the pandemic, all mental outcomes after short-term or acute stress response may also yield noteworthy findings.

## Conclusion

In summary, although further research is needed, this study represents an important step in exploring how stress of COVID-19 may be related to anxiety among Chinese college students. The results show that EI and MHL serve as three mechanisms by which stress of COVID-19 is all associated with anxiety. The effect of chain mediation provides positive implications showing that it may be coupled with stress of COVID-19 to further mitigate the onset of stress while relieving anxiety. Future research can help the field design targeted interventions for tackling specific areas of concern, such as the COVID-19 pandemic, as well as future issues to come.

## Data Availability Statement

The original contributions presented in the study are included in the article/supplementary material, further inquiries can be directed to the corresponding author.

## Author Contributions

All authors listed have made a substantial, direct and intellectual contribution to the work, and approved it for publication. YH: conceptualization, investigation, methodology, validation, formal analysis, writing—original draft, writing—review and editing, data curation, resources, supervision, and project administration. BY: conceptualization, investigation, methodology, validation, formal analysis, writing—original draft, writing—review and editing, data curation, and resources. JT: writing—original draft, writing—review and editing, software, visualization, and resources.

## Funding

This work was supported by Anhui' Major Online Teaching Reform Research Project (2020zdxsjg150), Department of Education of Anhui Province (SK2020A0908), and Department of Education of Anhui Province (gxbjzd75).

## Conflict of Interest

The authors declare that the research was conducted in the absence of any commercial or financial relationships that could be construed as a potential conflict of interest.

## Publisher's Note

All claims expressed in this article are solely those of the authors and do not necessarily represent those of their affiliated organizations, or those of the publisher, the editors and the reviewers. Any product that may be evaluated in this article, or claim that may be made by its manufacturer, is not guaranteed or endorsed by the publisher.
